# The Book World of Medicine and Science

**Published:** 1907-07-13

**Authors:** 


### THE BOOK WORLD OF MEDICINE AND SCIENCE.

"A Dictionary of Medical Diagnosis." By Henry Lawrence McKisack, M.D., M.R.C.P. (London). (Published by Baillière, Tindall, and Cox, London. Price 10s. 6d. net. Demy 8vo. Pages xii.+583, with 77 illustrations in the text.)

This book professes to be "A Treatise on the Signs and Symptoms observed in Diseased Conditions; for the use of Medical Practitioners and Students"; but it is more than this, for in addition to signs and symptoms it deals with a very large number of the laboratory methods of examining and testing blood, urines, fæces, pus, vomit, and so on. The word "signs" in connection with diseased conditions is capable of a wide interpretation, of course, but we are not sure that the practitioner would expect to find clinical laboratory methods included under it. He will consequently be all the better pleased with the book.

The dictionary is a descriptive compendium of five different groups of subjects, which may be summarised as follows:—

1. *Symptoms*, their various causes and their significance.
2. *Definitions* of all sorts of terms used in medicine.
3. *Physical signs* and their significance, including not only the ordinary physical signs to be noted in diseased states of the chest and abdomen, but also reflexes, and such signs as those of von Græfe, Mœbius, Romberg, Trousseau, and so forth.
4. *Clinical laboratory methods* and tests, with the points that can be learned from them.
5. Special articles, such as those upon *x*-rays in diagnosis, opsonins, and vaccine treatment.

The amount of matter that the author has condensed into so small a volume is truly wonderful. The whole is arranged in alphabetical order as far as possible, and there is a good system of cross reference. For example, when blood examinations are described under B, the methods of making the examinations, the significance of the various departures from normal that may be found, and the fallacies that are likely to arise, are fully dealt with in an article of thirty-five pages, and when one looks up "Opsonic power" under "O" one is referred to "Blood examination," where it is described. This plan makes the book much more readable than it would be were it a mere dictionary such as its name implies.

There is a fair index at the end of the volume, a moment's reference to which is enough to show that the author has tried hard to include everything, rarities as well as common things. Aphasia, Back, pain in, Clasp-knife reaction, Hæmatemesis, Poikilocytes, Urines, and so on are a few examples of the common things discussed. Whilst Anisocoria, Biot's respiration, Cryoscopy, Hemiagensia, Pentosuria, Wintrich's sign, and so on are a few headings of paragraphs which will indicate the kind of out-of-the-way things that the practitioner may gain useful information about if he consults this book.

Although we find the volume praiseworthy, and likely to supply a want, we are at the same time bound to draw attention to certain defects in it. We do not agree with the author in all the statements he makes. To give an example, he would lead the practitioner to suppose that a Widal's test was sufficiently positive to indicate typhoid fever if the reaction were complete in one hour with a dilution of the serum of only 1 in 50; whereas it has been clearly established that a positive Widal's test should be complete clumping within half an hour with serum diluted 1 in 200.

We have not space to point out other instances in which our views differ from those of the text before us, but we would draw attention to another failing—namely, errors of omission. It is true that it must have been extremely difficult to know what to include in and what to omit from the book, but in purchasing a volume which professes to be a dictionary of medical diagnosis in which many clinical methods are described, one is a little disappointed not to find any mention of tubercle bacilli nor of the Ziehl-Nelsen method of staining them, in the index. It is true that these are described under sputum in the text, but it takes some time to find this out; and when tubercle bacilli in urine are discussed there seems to be no account of one of the chief difficulties in connection with them—namely, their distinction from smegma bacilli. The spirochœta pallida is surely important as an aid to diagnosis, yet it is not mentioned. Raynaud's disease and Stokes-Adams' disease are mentioned and briefly described, but there is no similar mention of Banti's disease, Graves's disease, and so on, each of which might well have been shortly defined if the former are. If the dictionary defines some of the diseases which bear men's names it should define all, to be consistent. Signs which bear proper names, such as those of Babinski, Biermer, Biot, Ellis, Erb, and so on are discussed, but the list is incomplete. Ewart's sign, for example, is wanting, amongst others. If one looks up "Pancreas" or "Brain" in order to discover how to diagnose diseases of these, one does not find either of them in the index. Boas' reagent for testing for free hydrochloric acid in gastric juice is omitted; Cammidge's reaction for pancreatic disorders is not mentioned, so far as we can find, though its value, or its lack of value, is one of the things so much under discussion that many a doctor would like to read about it in some such book as this.

The shortcoming of the volume is mainly in the direction of incompleteness; this is a very grave fault in a dictionary, but it is one that can be remedied in another edition. On the other hand, it is a great boon to have by one a small volume in which are described not only all the common things, but also such matters as Haldane and Lorraine Smith's CO method of estimating the total volume of blood in the living body; the exact procedure in determining an opsonic index; the method of estimating the saline concentration of the serum, and so on; we feel sure that the book will be of value to the hospital physician as well as to the student and practitioner for whom it is ostensibly written.

